# Generation of three-dimensional optical cusp beams with ultrathin metasurfaces

**DOI:** 10.1038/s41598-018-27895-z

**Published:** 2018-06-22

**Authors:** Weiwei Liu, Yuchao Zhang, Jie Gao, Xiaodong Yang

**Affiliations:** Department of Mechanical and Aerospace Engineering, Missouri University of Science and Technology, Rolla, MO 65409 USA

## Abstract

Cusp beams are one type of complex structured beams with unique multiple self-accelerating channels and needle-like field structures owning great potentials to advance applications such as particle micromanipulation and super-resolution imaging. The traditional method to generate optical catastrophe is based on cumbrous reflective diffraction optical elements, which makes optical system complicated and hinders the nanophotonics integration. Here we design geometric phase based ultrathin plasmonic metasurfaces made of nanoslit antennas to produce three-dimensional (3D) optical cusp beams with variable numbers of self-accelerating channels in a broadband wavelength range. The entire beam propagation profiles of the cusp beams generated from the metasurfaces are mapped theoretically and experimentally. The special self-accelerating behavior and caustics concentration property of the cups beams are also demonstrated. Our results provide great potentials for promoting metasurface-enabled compact photonic devices used in wide applications of light-matter interactions.

## Introduction

Catastrophe theory mathematically describes the formation and dramatic change of bifurcations as geometrically stable structures in nonlinear potential functions under the perturbations of external control parameters^1,2^. Catastrophe theory bridges geometry interpretation to the physical world and its manifestation in optics appears in high-intensity caustics with defined geometries of different orders^3–5^. According to the increasing dimensionality of the control parameter space, seven elementary catastrophes have been hierarchically classified including four cuspoid catastrophes (fold, cusp, swallowtail, butterfly) and three umbilic catastrophes (hyperbolic, elliptic, parabolic)^1,2^. Subsequent to the Airy beam with typical fold caustics which is the first realized optical catastrophe^6,7^, the regular polygon beam (RPB) emerges as a paraxial beam with multiple cusp points equally spaced in a circle and propagating along a bending trajectory. The triple RPB corresponds exactly to the elliptic umbilic catastrophe^8–11^. In contrast to the fold caustics, this kind of cusp caustics owns not only the simultaneous self-healing and self-bending propagation properties but also has the remarkable caustics concentration property which leads to the generation of needle-like field structure^8,12–14^. Such novel beam properties of cusp beams will enable many spectacular applications such as optical particle micromanipulation and transportation^15,16^, super-resolution imaging^17,18^ and curved plasma channels^19^. Optical catastrophe is traditionally generated by using reflective diffraction optical elements like SLM (spatial light modulator)^6,16^ or DMD (digital mirror device)^12^. However, these bulky devices usually have micrometer pixel size and the limited working-wavelength range, which inevitably hinder the further exploration of optical catastrophe integrated in miniaturized photonic devices and systems.

Recently, the manipulation of complex light field is getting rapid growth with the development of ultrathin geometric phase based metasurfaces^20,21^. Different from bulky optical components with the phase shift depending on the optical path, metasurfaces can introduce the Pancharatnam-Berry geometric phase accompanied with polarization conversion, which can overcome narrow bandwidth limitation and make the optical device compact^22–27^. Nowadays metasurfaces have been widely designed to shape the phase front of light field in many applications such as structured beam generation^28–35^, flat lens imaging^36–39^, compact wave plates for polarization conversion^40–42^ and holographic image construction^43–47^.

In this work, we investigate and analyze the generated 3D cusp beams with geometric phase based ultrathin plasmonic metasurfaces constructed from nanoslit antennas. The Pancharatnam-berry (PB) geometric phase profile encoded in the metasurface is taken from the Fourier spectrum of cusp beam, and the local geometric phase is realized by varying the orientation angle of nanoslit antenna in each unit cell. Here three kinds of cusp beams with different numbers of self-accelerating channels have been studied. We have analyzed the whole beam propagation profile of cusp beams accompanied with their novel self-accelerating property and caustics concentration property at three different wavelengths of 633 nm, 808 nm and 980 nm, covering a broadband wavelength range. Furthermore, it is shown that the generated 3D cusp beams from the metasurface gets robust self-healing property over obstacles during propagation. Our demonstrated results will provide promising possibilities for building metasurface-enabled ultrathin photonic devices used in many applications of light-matter interactions such as complex light field conversion, optical particle manipulation, and super-resolution imaging.

## Results

### Design of ultrathin plasmonic metasurfaces

**Figure 1:**
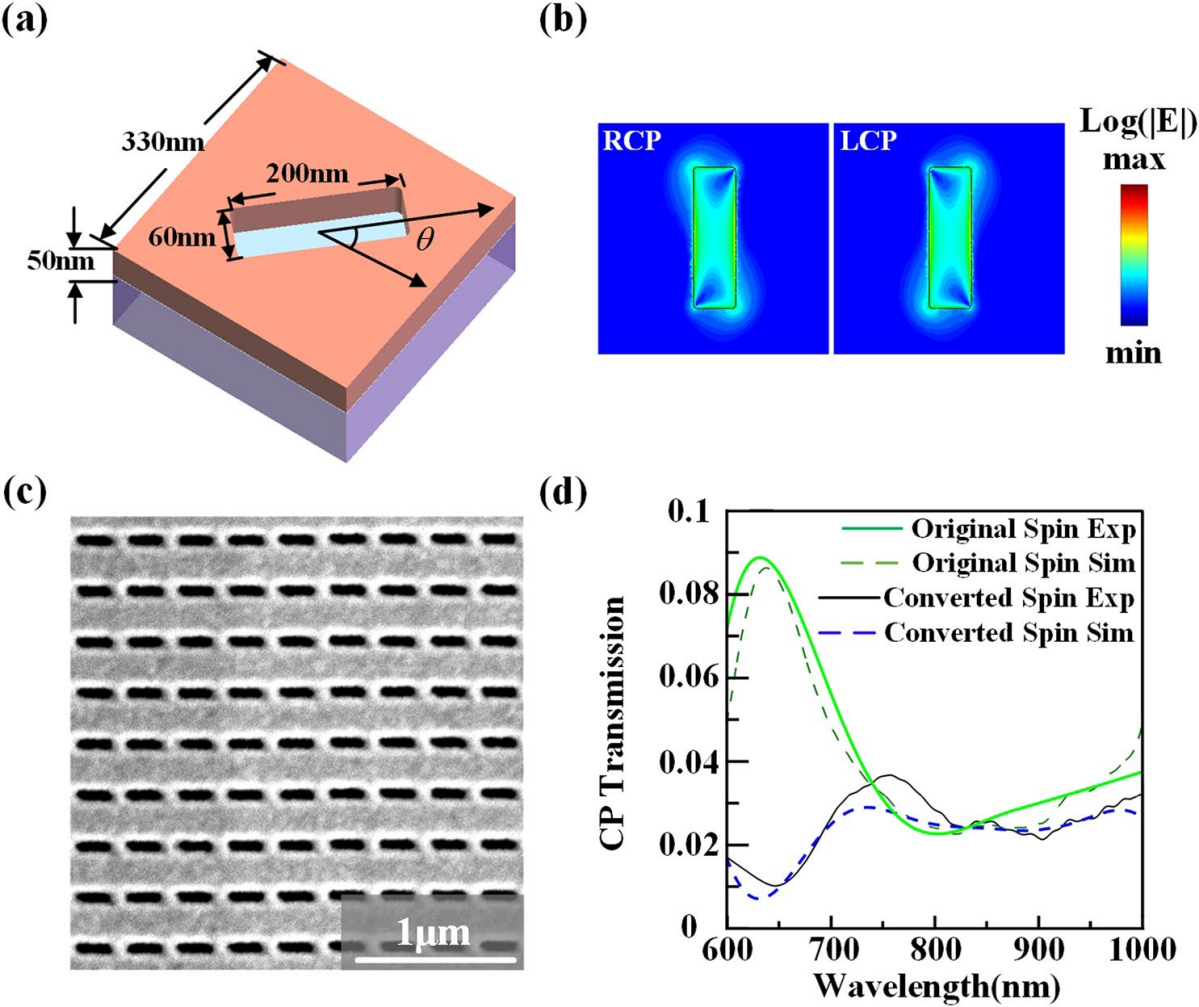
(**a**) Schematic of the unit cell design of the nanoslit antenna at the orientation angle of *θ*. (**b**) Simulated electric field log|*E*| distributions of the nanoslit antenna under circular polarization basis at the wavelength of 800 nm in the middle plane of nanoslit. (**c**) A SEM image of the fabricated homogeneous nanoslit antenna array. (**d**) Measured and simulated transmission spectra under circular polarization basis.

The designed plasmonic metasurface based on the PB geometric phase with compact size of 50 μm by 50 μm contains 150 by 150 subwavelength nanoslit antennas with specified orientation angles. As shown in Fig. 1a, the nanoslit antenna is etched into a thin gold film with thickness of 50 nm on glass substrate, the width and length of each nanoslit antenna is 60 nm and 200 nm respectively, and the period of the unit cell is 330 nm. The PB geometric phase introduced by each nanoslit antenna is only determined by the antenna orientation angle *θ*. When the incident circularly polarized beam normally transmits through the anisotropic nanoslit antenna, the original spin component has no phase shift, while the converted spin component will have the induced geometric phase shift of 2*θ* based on the general principle of Pancharatnam-Berry optical elements (PBOEs)^23–25,33^. The overall phase distribution from the metasurface is obtained by arranging the nanoslit antennas into the designed spatially inhomogeneous array. Figure 1b plots the electric field distributions |*E*| under circular polarization basis (left-handed and right-handed circular polarizations, LCP and RCP), showing the polarization anisotropy of the nanoslit antenna. A SEM image of the fabricated homogeneous array of nanoslit antennas is shown in Fig. 1c. Figure 1d shows the measured and simulated transmission spectra from the nanoslit antenna array under circular polarization basis. In this case, the original spin component is chosen as LCP, and the converted spin component is RCP. It is found that the simulated results match well with the experimental results, proving the effectiveness of the designed nanoslit antennas.

Recently the class of RPBs has been extended with both odd and even numbers of cusp points, and such accelerating cusp beams with four or five cusp points are derived from Thom’s elliptic umbilics. These cusp beams naturally focus in free space to yield caustics with a non-trivial morphology that does not correspond to those described by standard catastrophe optics^8,9,11^. Here the targeted geometric phase profiles for generating the cusp beams with three different numbers of self-accelerating channels are determined by the beam Fourier spectra. The integral representation for the electric field of cusp beams in the Cartesian coordinates can be written as^8,9,11^

1 $$E({x},{y},{\rm{z}})=A{\int }_{-\infty }^{+\infty }{\int }_{-\infty }^{+\infty }{\exp }[ik{\psi }({\xi },{\eta },{x},{y},{z})]{\rm{d}}\xi {\rm{d}}\eta $$

The phase function in the integral can be written as *ψ*$(\xi ,\eta ,\,x,y,z)$ = ${\varphi }_{m}({\xi },{\eta })-z({\xi }^{2}+{\eta }^{2})-({\xi }x+{\eta }y)$, *A* is a constant and ${k}=2\pi /\lambda$ is the wave number. $({\xi },{\eta })$ denote the transverse coordinates in the input plane and $(x,y,z)$ represent the coordinates in the propagation plane, and the generation of cusp beams is realized by the Fourier transform for the phase term ${\varphi }_{m}({\xi },\,\eta )$^8,11^. Then the orientation angles $\theta$ of nanoslit antennas in metasurface is determined by $\theta ({\xi },\eta )$ =$\,{\varphi }_{m}({\xi },\,\eta )/2$. Through the coordinate transformation, the general expression of $\,{\varphi }_{m}({\xi },\,\eta )\,$in the polar coordinate can be written as^10^

2 $$\,{\varphi }_{m}({R},{\theta })=C{R}^{m}\,{\sin }(m{\theta }+w)$$

where $({R},{\theta })$ are the polar variables in radial and azimuthal directions, *C* and *w* represent a constant scaling number and the initial phase, respectively. Specially, the polynomial order m determines the number of self-accelerating channels with the intensity maximum of the cusp beams.

**Figure 2:**
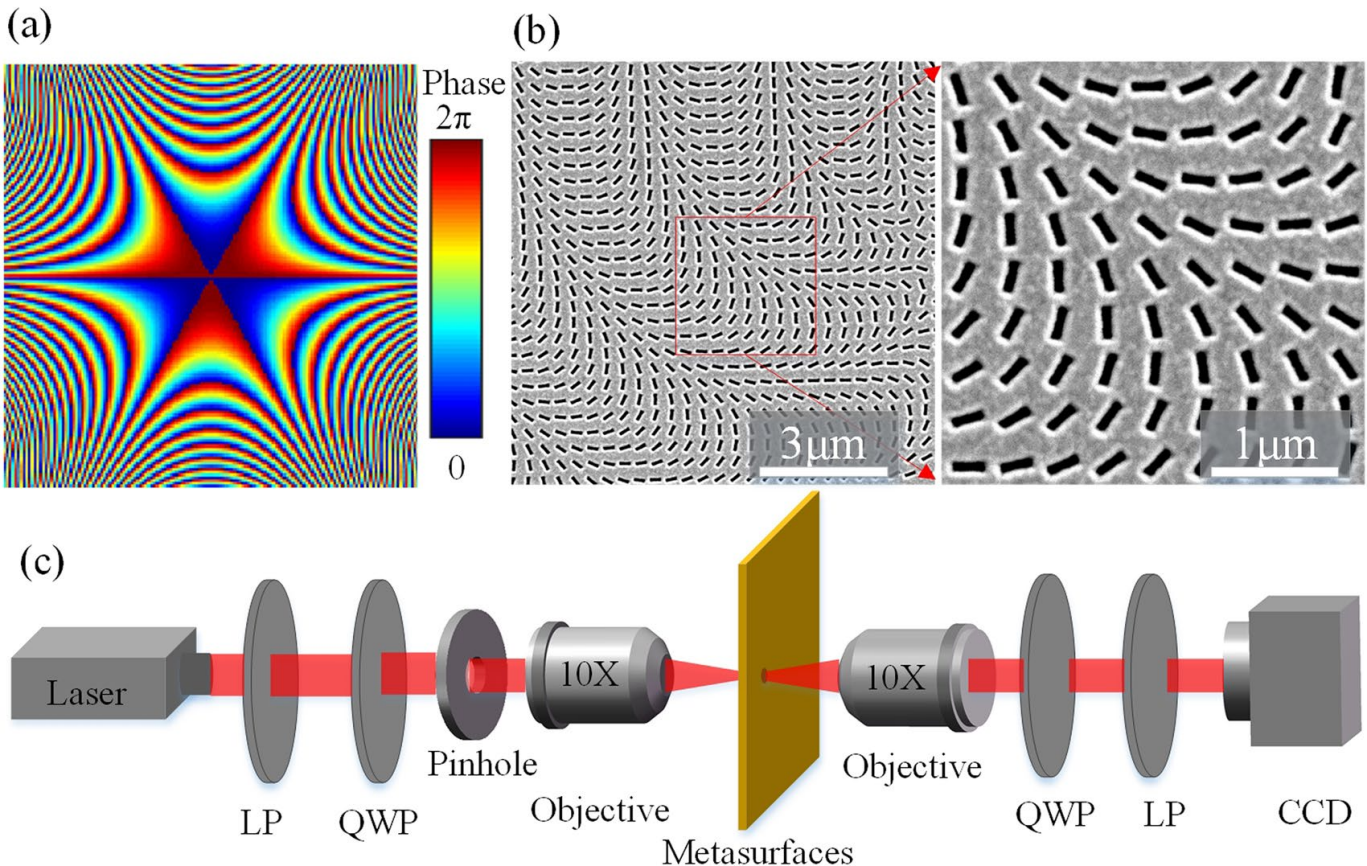
(**a**) The phase profile to generate the optical cusp beam with *m* = 3, *C* = 3.3 and *w* = $\,{\rm{\pi }}/2$. (**b**) SEM images for the fabricated metasurface. (**c**) Schematic of the experimental setup.

In order to illustrate the special characteristics of cusp beams having various number of self-accelerating channels, three metasurfaces with *m* = 3, 4, 5 are designed. Figure 2a shows the typical phase profile to generate the optical cusp beam with *m* = 3, *C* = 3.3 and $w={\rm{\pi }}/2$. The SEM images for the fabricated metasurface are shown in Fig. 2b. Figure 2c describes the scheme of experimental setup, where a circular polarized incident beam from a laser diode is prepared through a linear polarizer and a quarter-wave plate. Then a pinhole with diameter of 500 μm is imaged upon the metasurface by a 10× objective lens. Another 10× objective lens is used for the Fourier transform of the transmitted beam out from the metasurface. Here another set of a quarter-wave plate and a linear polarizer is used to select out the generated 3D cusp caustics in the converted spin component. A CCD camera is placed on a translation stage to record the 3D intensity profile of the produced optical cusp beams.

### Generation of optical cusp beams with special beam structures

**Figure 3:**
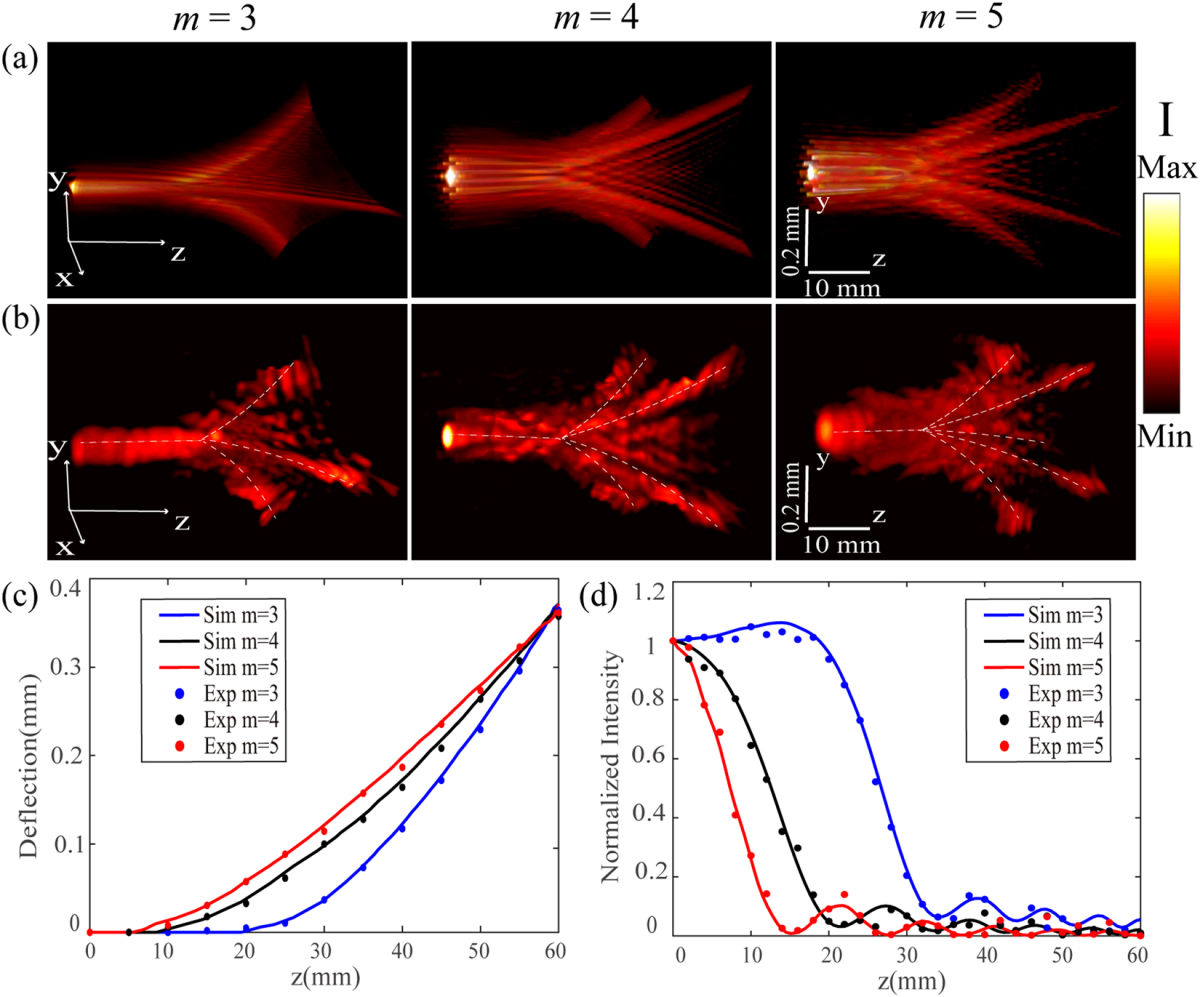
(**a**) Calculated and (**b**) experimental 3D intensity structures for optical cusp beams with *m* = 3, 4, 5. (**c**) The beam deflection of self-accelerating channels during the propagation and (**d**) the optical intensity distribution along the optical axis for three cusp beams.

**Figure 4:**
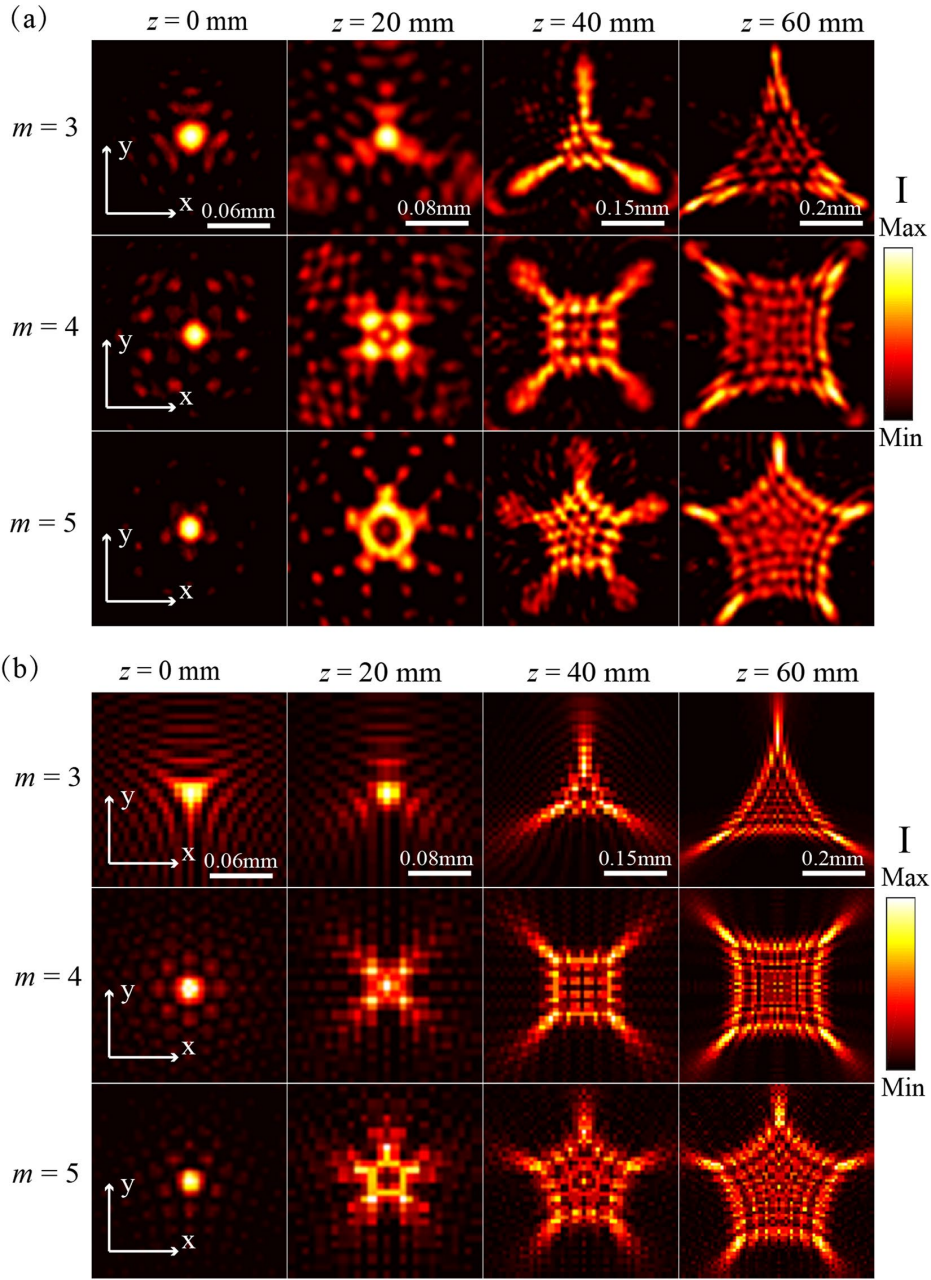
(**a**) Measured (**b**) calculated transverse optical intensity profiles of the generated cusp beams at several *z* positions at the wavelength of 633 nm. Figures in each column share the same scale bar.

The special beam structures of optical cusp beams with *m* = 3, 4, 5 are first measured at the wavelength of 633 nm. In order to keep the similar beam sizes for the generated optical cusp beams with different *m*, the initial phase is $w={\rm{\pi }}/2\,$and the scaling number for *m* = 3, 4, 5 is set to be *C* = 3.3, 1.2, 0.52 respectively. Figure 3a displays the calculated 3D intensity |*E*|^2^ skeleton of cusp beams with *m* =  3, 4, 5 based on Eq. (1) and Eq. (2), and the corresponding reconstructed 3D intensity structure in experiment is shown in Fig. 3b. It is shown that the cusp beams exhibit the unique 3D beam structures with multiple self-accelerating channels and the needle-like concentrated optical field. Distinguished from the bending beam structure of Airy beam, the cusp beam shows the straight needle-like field in the front section of beam, here the needle-like field propagates straightly in the beam’s front section, where the *z* = 0 plane starts at the initial plane of Fourier transform in the spatial spectrum space. Then the needle-like field expands to multiple cusp lobes with the number of m which move away from the optical axis along the curved paths, showing the self-accelerating property during the beam propagation. Such unique beam structure of cusp beams exhibits great potentials in applications such as optical particle manipulation and super-resolution imaging. Furthermore, the beam deflection of self-accelerating channels during the propagation and the optical intensity distribution along the optical axis for the above three cusp beams are compared in Fig. 3c,d, respectively. It is observed that the length of needle-like field is inversely proportional to the parameter *m*, and the beam deflection angle has the similar tendency and becomes identical at the position of *z* = 60 mm. At *z* > 60 mm, the cusp beams will still keep the transverse cusp intensity profiles but with enlarged sizes and weaker intensity as the propagation distance increases. In order to further reveal the beam structure of cusp beams, Fig. 4a,b show the measured and calculated transverse optical intensity profiles of the generated cusp beams at several *z* positions during the beam propagation at the wavelength of 633 nm, showing good consistency with each other. It is observed that a focused region with intensive optical intensity exists in the beam center at *z* = 0 plane showing the transverse structure of the needle-like field for each cusp beams. Afterwards, the transverse intensity profiles during the propagation are evolved into multiple cusp points from the initial central singular point. It is observed that each cusp beam can keep the stable cusp intensity pattern for long distance but with the enlarged size during propagation, indicating the shape-invariant and robust propagation property for the cusp caustics generated by the metasurfaces. From the beam pattern, it can be seen that the intensity distribution for the generated cusp beam of *m* = 3 is the typical elliptic umbilic catastrophe^48^, while the beam patterns for cusp beams of *m* = 4, 5 are consistent with the previous results from RPBs^10^, which can provide variable numbers of optical trapping channels for particle transportation.

**Figure 5:**
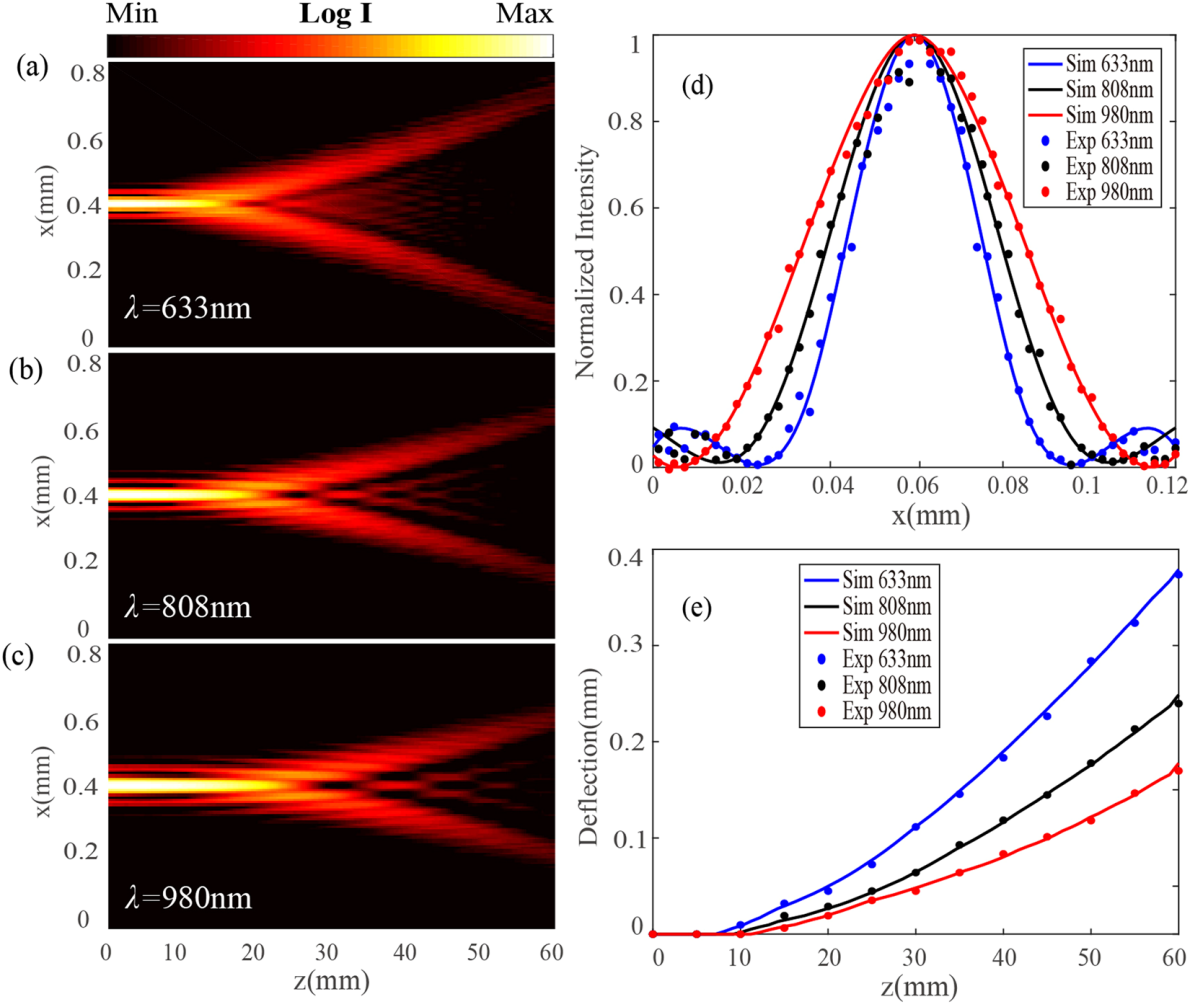
(**a–c**) Calculated optical intensity profiles in x-z plane for the cusp beams with *m* = 4 at 633 nm, 808 nm, 980 nm. (**d**) Calculated and measured transverse intensity distribution at *z* = 0 plane. (**e**) Calculated and measured beam deflection along the curved cusp lobes during the propagation.

Broadband generation of stable-shape cusp caustics with the designed metasurface will further expand their applications. The calculated optical intensity profiles in *x-z* plane for the optical cusp beam with *m* = 4 at three wavelengths of 633 nm, 808 nm, 980 nm are plotted in Fig. 5a–c. In the calculation, the parameter setting for the cusp caustics is the same as the above design but with *w* = 3*π*/2 to rotate two cusp points on the *x* axis. It is shown that the longitudinal length and transverse width of the needle-like field for the cusp beam get larger as the operation wavelength increases, but the beam deflection of the evolved cusp lobes from the needle-like structure has the inverse proportion to the increased wavelength. Additionally, Fig. 5d,e plot the transverse optical intensity distribution at *z* = 0 plane and the beam deflection along the curved cusp lobes in both calculation and experiment, which verify the above beam properties of the cusp caustics influenced by the operation wavelength.

**Figure 6:**
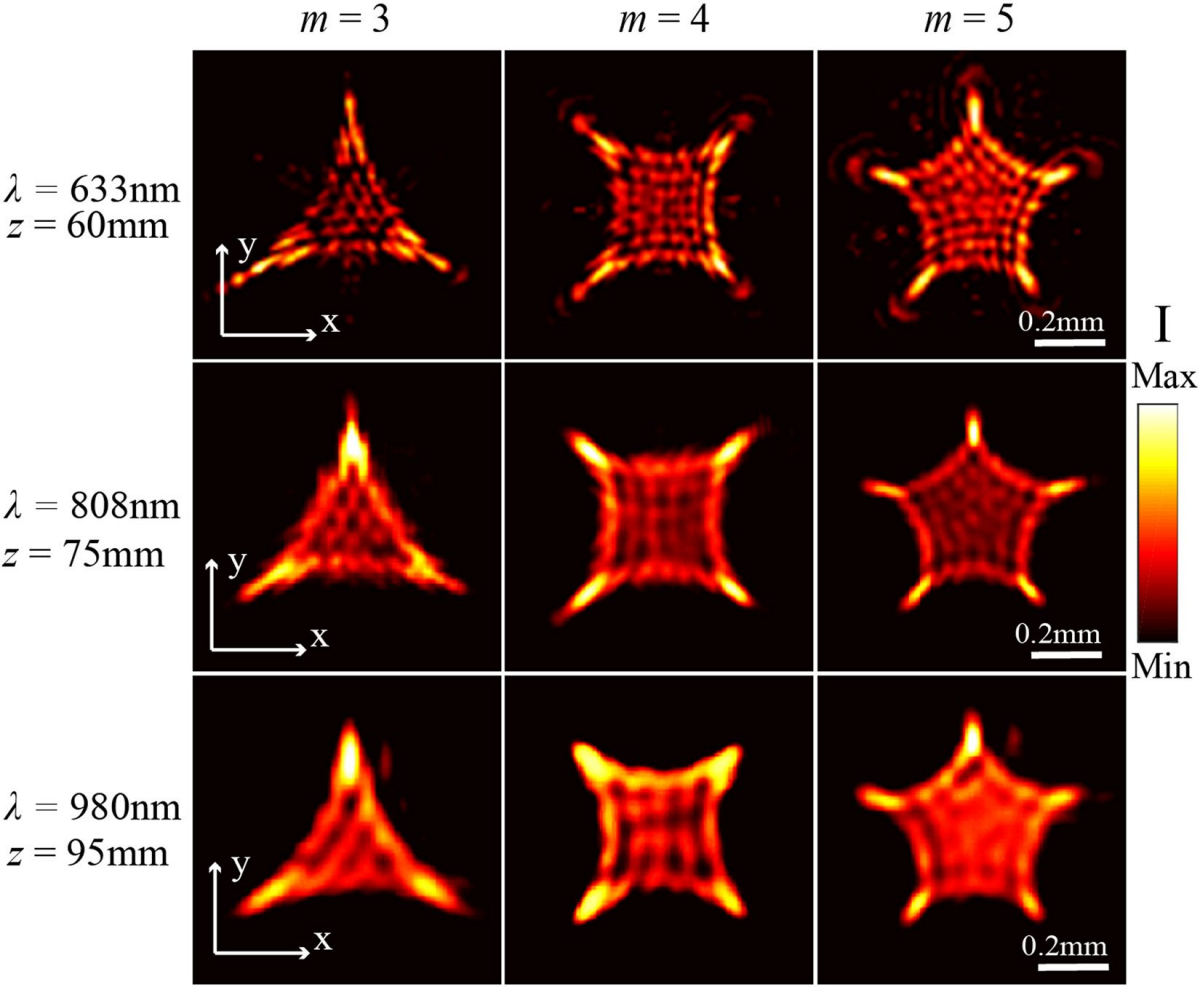
Measured transverse intensity patterns for cusp beams with *m* = 3, 4, 5 at the wavelengths of 633 nm, 808 nm, 980 nm in the propagation planes of *z* = 60 mm, 75 mm, 95 mm, respectively.

Figure 6 presents the measured transverse intensity patterns for optical cusp beams with *m* = 3, 4, 5 generated from the metasurfaces at the three wavelengths in the propagation planes of *z* = 60 mm, 75 mm, 95 mm, respectively. It is seen that the beam sizes and optical intensity patterns for each cusp beam at the three wavelengths are almost the same, but the longer propagation distance is needed to get the stable multiple cusp points at the longer operation wavelength. At one certain wavelength, the three cusp beams at the same propagation plane get the similar beam deflection for the cusp lobes.

### Self-healing property of optical cusp beams

**Figure 7:**
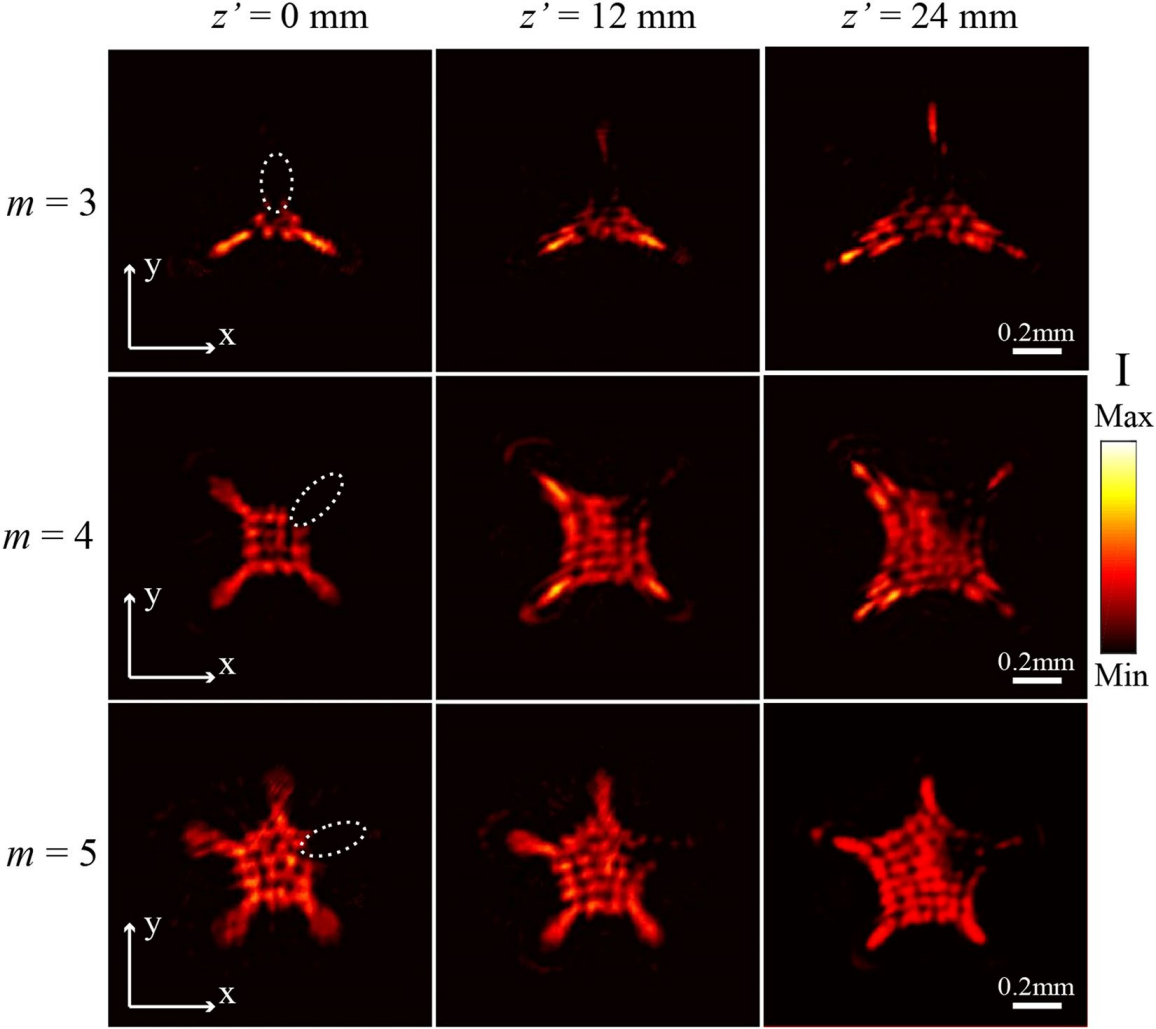
Self-healing property of the generated optical cusp beams with *m* = 3, 4, 5 at 633 nm.

According to the catastrophe theory, optical cusp beams with stable cusp caustics formed from the nonlinear potential function occupies the minimal potential energy, indicating robust self-accelerating geometrical structures^8,11^. In order to experimentally demonstrate the structural stability of the cusp beams generated by the metasurface at the wavelength of 633 nm, a small opaque obstacle is employed to completely block one arbitrary cusp point and then the self-healing property of optical cusp beam during the propagation is monitored. The blocked beams with *m* = 3, 4, 5 right after the obstacle are shown in the first column of Fig. 7, where this position is defined as the initial measurement plane *z*’ = 0. Behind the obstruction, it is seen that the blocked cusp points of all the three cusp beams gradually recover themselves with the increasing propagation distance. Concretely, at about *z*’ = 12 mm, the weak cusp points appears in the blocked arms as shown in the second column of Fig. 7. At the propagation plane *z*’ = 24 mm, all the cusp beams can reconstruct the blocked points with the whole cusp shapes appearing as shown in the third column of Fig. 7. It is noted that the optical intensity of the recovered cusp points is weaker than the unblocked cusp points, which is caused by the energy loss resulted from the blocking. After all, it is demonstrated that the optical cusp beam generated by the metasurface also has the special self-healing propagation property. Such beam property may further promote wide applications of optical cusp beams such as particle manipulation and biology tissue imaging, where the light absorption or scattering problems may be overcome to some extent because of the self-construction property of the cusp beams.

## Discussion

In summary, the generation of 3D optical cusp beams with different numbers of cusp lobes has been realized by using ultrathin plasmonic metasurfaces based on geometric phase in a broadband wavelength range. The special 3D beam structures of cusp beams with the needle-like fields and multiple self-accelerating channels have been demonstrated at multiple wavelengths. Furthermore, the experimental results indicate that the cusp caustics generated from the designed metasurface also own robust self-construction property over obstacles during the propagation. Our demonstrated results will find many promising applications in light-matter interactions such as particle trapping and transport, super-resolution imaging, and laser nanomanufacturing.

## Methods

### Simulation

The simulations in Fig. 1 are conducted by using the CST microwave studio software. The permittivity of gold is taken from spectroscopic ellipsometry data fitted with a general oscillator model and the refractive index of glass substrate is 1.45. The unit cell is enclosed by periodic boundary conditions at both *x* and *y* directions.

### Sample preparation

A 50 nm-thick gold film is deposited on a glass substrate using electron-beam evaporation. Then the designed antenna arrays are milled in the gold film using focused ion beam (FIB) system (FEI Helios Nanolab 600, 30 kV, 9.7 pA). To achieve the targeted metasurface, the orientation angle for the nanoslit antenna in each unit cell is determined by the designed phase profile for the cusp beam.

### Optical characterization

To achieve the transmission spectra measured from the metasurface shown in Fig. 1 under circular polarization basis, a collimated broadband Tungsten-Halogen source is employed with the combination of a linear polarizer and an achromatic quarter-wave plate which can convert the incident light to circularly polarized wave. A 50× objective lens is used to focus the light beam normally onto the metasurface and then the transmitted light is collected by another 10× objective lens to a spectrometer (Horiba, iHR 550). Another set of a quarter-wave plate and a linear polarizer is used to separate the transmitted converted spin component through the metasurface. Here a transparent glass substrate is utilized to normalize the transmission spectra.
